# Participation in leisure-time activities among people living with Spinal Cord Injuries. A cross-sectional survey

**DOI:** 10.1038/s41394-025-00722-0

**Published:** 2025-10-23

**Authors:** Frederik Skovbjerg, Henriette Holm Stabel, Matthijs Ferdinand Wouda, Peter William Stubbs, Jørgen Feldbæk Nielsen

**Affiliations:** 1 Spinal Cord Injury Center of Western Denmark, Viborg Regional Hospital, Viborg, Denmark; 2 Department of Clinical Medicine, Faculty of Health, Aarhus University, Aarhus, Denmark; 3 Hammel Neurorehabilitation Center and University Research Clinic, Hammel, Denmark; 4 Sunnaas Rehabilitation Hospital, Nesodden, Norway; 5 Oslo Metropolitan University, Department of Rehabilitation Science and Health Technology, Oslo, Norway; 6 Discipline of Physiotherapy, Graduate School of Health, University of Technology Sydney, Sydney, Australia

**Keywords:** Spinal cord diseases, Population screening, Quality of life

## Abstract

**Study design:**

Cross-sectional descriptive survey.

**Objectives:**

To assess participation levels, types of leisure-time activities, and barriers to engagement among people with spinal cord injury (SCI), and to identify demographic and functional factors influencing participation.

**Setting:**

Western Denmark.

**Methods:**

Between September and November 2023, adults with SCI were invited to complete a digital survey. Inclusion criteria were any level of SCI. Data collected included demographic and injury-related characteristics, types and frequency of leisure-time activities, social context, and perceived participation barriers. Analyses involved descriptive statistics, prevalence proportions, and prevalence proportion ratios.

**Results:**

Of 1259 eligible persons, 479 completed the survey. Participants engaged in a median of three different leisure-time activities over the past year. In the previous four weeks, 19% reported participating in leisure activities less than once per week. Participation in non-social and social activities less than once per week was reported by 44 and 37%, respectively. Lower participation was more common among persons with shorter educational attainment and reduced mobility at 100 meters. Most activities were self-organized or provided by commercial providers. The most commonly reported barriers were physical limitations, time constraints, and activity suitability.

**Conclusions:**

Participation in leisure-time activities among people with SCI varies widely. In this study, social engagement, education level, and mobility were observed to be related to patterns of participation. Addressing physical, contextual, and logistical barriers through tailored interventions may enhance leisure-time engagement and support more holistic rehabilitation outcomes.

## Introduction

Spinal cord injury (SCI), whether of traumatic or non-traumatic origin, often results in profound and enduring changes in an individual’s health status and functional capacity [1, 2]. Irrespective of the underlying cause, individuals with SCI typically undergo a period of subacute rehabilitation prior to returning to their home environments. The primary objective of rehabilitation is to optimize participation in daily life activities, which is essential for enhancing overall quality of life [3]. Although functional improvements are generally observed during rehabilitation, outcomes vary considerably due to factors such as the severity of the injury, access to healthcare services, individual resources, and support from family or caregivers [4]. Following SCI, individuals often experience changes in their living arrangements. The transition from hospital-based rehabilitation to home is a pivotal stage, frequently accompanied by challenges that require adaptation in terms of participation, functional ability, and daily engagement [5]. The physical home environment, as well as the ability to manage both pre-existing and new routines, roles, and social interactions, significantly affects the success of this transition [6–9]. Higher levels of activity and participation are positively associated with increased life satisfaction [10, 11]. Despite comprehensive rehabilitation efforts, many individuals with SCI continue to experience limitations in performing activities of daily living (ADLs), maintaining autonomy, and engaging fully in community life [12]. Barriers to community participation may be both personal and environmental in nature [7, 13]. Community participation encompasses a broad spectrum of involvement in social, organizational, and professional contexts, whether through employment or voluntary activities. It is recognized as a critical determinant of health and well-being, contributing to individuals’ sense of belonging, identity, and purpose. For individuals with SCI, active engagement in community life can mitigate social isolation, promote psychological resilience, and support the maintenance of functional abilities [14, 15]. Furthermore, community participation is closely linked to broader societal inclusion and equity, highlighting its relevance as both a clinical outcome and a public health concern [16, 17]. Among these, employment and return-to-work processes have received considerable attention within both clinical and research domains [18, 19]. In contrast, participation in leisure-time activities remains relatively underexamined. These activities, often embedded in community contexts and characterized by shared interests and social interaction, are particularly salient in Northern European cultural settings. A more detailed understanding of leisure participation could yield important insights for supporting active and meaningful lives among individuals with SCI. This study aims to examine patterns of participation in leisure-time activities among persons living with spinal cord injury (SCI) in Denmark. Specifically, it investigates both the perceived barriers that constrain engagement and the motivation levels. By identifying these elements, the study seeks to contribute evidence that can inform the design of targeted rehabilitation interventions and guide the development of inclusive, community-based leisure opportunities. Such insights are intended to support long-term social integration and enhance quality of life for individuals with SCI.

## Methods

### Study design

This study was designed as a descriptive cross-sectional study based on digital surveys conducted from September 2023 to November 2023. The study was approved by the local ethical committee of the Central Denmark Region (reference number: 1-45-70-60-23). All methods were performed in accordance with the relevant guidelines and regulations. Informed consent was obtained from all participants, who confirmed their willingness to participate by completing the digital survey. Data were stored and managed using REDCap electronic data capture tools [20] hosted at Aarhus University. The STROBE guidelines were used for reporting results [21].

### Participants

We included persons who were ≥18 y/o and had been in contact with the Spinal Cord Injury Center of Western Denmark (SCIWDK). Participants were identified through the electronic medical records database of patients who had been admitted to SCIWDK within the last 10 years, as well as from a local research database that included patients who had been in contact with SCIWDK before 2013. Eligible participants were contacted through secure digital mail using their Danish social security numbers and were invited to complete an online survey. If participants did not respond to the initial invitation, reminders were sent twice, at intervals of 2 and 4 weeks. The invitation also provided an offer from the first author to assist in the technical completion of the survey.

### Data sources

Age and biological sex were derived from the participants’ social security numbers, while all other data were obtained from the digital survey.

### Questionnaire

The questionnaire included questions on demographics, characteristics of SCI, participation in leisure-time activities, and motivation and barriers to leisure-time participation. The section on participation was modified from a previous study on sports and hobbies in the Danish general population [22] where participants indicated the activities participated in and the frequency of participation in each activity. These items were selected because they were originally developed in Danish, culturally appropriate for the Danish context, and had previously been applied in populations including people with disabilities. To ensure their suitability for individuals with SCI, the wording of selected questions was adjusted to reflect different functional levels (e.g., use of a wheelchair). The adapted questionnaire was then reviewed by 10 individuals with SCI, whose feedback was used to refine the wording further prior to data collection.

### Demographic and injury related characteristics

Participants provided data on work status, education level, living situation, SCI characteristics, functional level, year of SCI, and place of living. Age was categorized into 18-30, 31-45, 46-60, 61-76, and 76 + . Work status was classified into employed, retired, early retired, and other. Education levels ranged from primary school to higher education and were classified into secondary education, vocational education, short education ( < 3 years), intermediate education (3-4 years) and higher education ( > 4 years). Living situation was classified as living alone or living with someone. Functional level was assessed using item 13 from the Spinal Cord Independence Measure (SCIM), regarding ambulation over moderate distances (100 m), with options for wheelchair use, walking aids, or unassisted walking [23]. SCIM item 13 responses were grouped into: using a wheelchair with assistance, using a manual wheelchair without assistance, walking with aids, and walking without aids. Years since injury was categorized into 0-2, 3-5, 6-10, 11-15 and 16+ years. Postal codes were classified as urban or rural.

### Participation in leisure-time activities

The questionnaire assessed the type, frequency, and context of leisure-time activities. Participants selected from 64 possible activities, which they engaged in over the past 12 months, providing an overview of their activity repertoire. For each reported activity, they were then asked about their participation during the past 4 weeks to capture more recent and reliable information on activity frequency. Response options ranged from *more than five times per week* to *less than once per week*. Participants also indicated whether they usually engaged in the activity alone or with others. If with others, they specified the context, such as voluntary associations, commercial providers, self-organized groups, or other community-based groups. The participation score, which acts as a proxy for leisure-time participation, considers both the number and frequency of leisure-time activities. Separate scores were calculated for activities performed alone (non-social) and with others (social), as well as a combined total score. Participation levels were categorized into ‘<1 per week’, ‘~1 time per week’, ‘~2 times per week’, ‘~3-4 times per week’, ‘≥ 5 times per week’. For a detailed description of the score, see Supplementary material S.2.

### Motivation and barriers

Participants were asked how motivated they were to engage more in leisure-time activities than they currently are, with response options ranging from not at all motivated to highly motivated. Lastly, participants were asked to indicate barriers hindering participation in leisure activities, with choices including lack of time, physical limitations, financial constraints, or difficulties in finding suitable activities or support.

### Statistics

Demographic characteristics were summarized using medians, minimum and maximum values as data were non-normally distributed. Categorical variables we presented as proportions. We performed a complete-case analysis, excluding participants with incomplete data from all relevant variables. In the analysis of participation in different leisure-time activities within the last 12 months, we presented data as medians with 95% confidence intervals (CI 95%) calculated using bootstrap resampling [24] both un-stratified and stratified on relevant variables. In the analysis of participation within the last 4 weeks, we presented prevalence proportions (PP) with CI 95% of participation frequency for total, non-social and social participation patterns. Further, we presented prevalence proportion ratios (PPR) stratified by demographic and SCI related characteristics to describe associations with participation <1 time per week. CI 95% accompanying proportions and ratios were calculated using Wilsons method [25]. The organization of the leisure-time activities, and participants’ motivation were presented as proportions, while the perceived barriers were presented as frequencies. We used Python with the package pandas, numpy, and stat. for all processing and analysis of data.

## Results

We identified 1259 eligible participants. Of these 102 (8%) did not have a secure e-mail address hence 1157 were invited to participate. Of these, 563 (45%) initiated the questionnaire and 479 (38%) completed it. The participants’ demographic and injury related characteristics are presented in Table 1.

**Table 1:** Demographic and injury-related characteristics of the participants (n = 563).

Characteristics	n	Value
**Biological sex**	563	
Male	369	66%
Female	194	34%
**Age, median (min/max)**	563	62 (23/94)
**Work status, %**	511	
Working	160	31%
Retired	170	33%
Early retiree	158	30%
Under education	5	1%
Unemployed	13	3%
On leave	5	1%
**Educational level, %**	511	
Primary school	71	14%
Secondary education	13	3%
Vocational education	153	30%
Short (Under 3 years)	53	10%
Intermediate (3-4 years)	135	26%
Higher education (Over 4 years)	80	16%
Other education	6	1%
**Living situation, %**	511	
Living alone	156	30%
Living alone with children	22	4%
Living with partner without children	188	37%
Living with partner with children	131	26%
Living with parents	4	1%
Living with roommates	3	1%
Other	7	1%
**SCI characteristics, %**	504	
Paraplegia	256	50%
Tetraplegia	235	47%
Other	13	3%
**Functional level at 100** **m, %**	503	
Walking without assistance	156	31%
Walking with difficulties	140	28%
Self-sufficient in manual wheelchair	120	24%
Electric wheelchair or partial assistance in manual wheelchair	61	12%
Wheelchair and full assistance	26	5%
**Years since injury, median (min/max)**	494	7 (1/56)

Values are presented as number and percentage (n (%)), unless otherwise stated. Age and years since injury are reported as median values with minimum and maximum in parentheses. The total number of participants varies across categories due to missing data. SCI; Spinal Cord Injury.

### Participation in different leisure-time activities within the last 12 months

Out of 563 participants, 535 completed the questionnaire on leisure-time activities within the last 12 months. Overall, the study population participated in 3 CI 95% (3.0;3.0) different activities. Table 2 shows the median number of leisure-time activities stratified by relevant variables. The findings suggest that participation in different leisure-time activities was higher within younger age groups and among people with higher functional levels. Higher numbers of participation in different activities were observed among people who were employed, in comparison to those who were early retired or retired. For other variables, including years since injury, living situation, and living area, no clear differences in leisure-time participation were observed between categories. The distribution of participation across specific activities is presented in Figure S1 in the supplementary material.

**Table 2:** Median (95% CI) number of participations in different leisure activities within the last 12 months stratified by relevant variables.

Variable	Categories	Median (95% CI)
**Biological sex**	Men (n = 352)	4 (4.0;4.0)
	Women (n = 183)	4 (4.0;5.0)
**Age groups**	18-30 (n = 15)	7 (4.0;10.0)
	31-45 (n = 61)	4 (4.0;7.0)
	46-60 (n = 156)	4 (3.0;4.0)
	61-76 (n = 217)	3 (2.0;3.0)
	76+ (n = 86)	2 (2.0;3.0)
**Work status**	Employed (n = 159)	4 (4.0; 5.0)
	Early retirement (n = 158)	3 (3.0; 3.0)
	Retirement (n = 167)	2 (2.0; 3.0)
	Other (n = 18)	3 (2.0; 6.0)
**Educational level**	Secondary education (n = 90)	2 (3.0;3.0)
	Vocational education (n = 206)	3 (2.0;3.0)
	Medium education (n = 135)	4 (3.0;4.0)
	Higher education (n = 80)	5 (4.0;6.0)
**Functional level at 100** **m**	Low (n = 86)	2 (1.5; 3.0)
	Intermedium (n = 119)	3 (3.0; 4.0)
	Medium (n = 139)	3 (2.0; 3.0)
	High (n = 155)	4 (3.0; 5.0)
**Time since injury**	0-2 years (n = 7)	5 (4.0; 9.0)
	3-5 years (n = 129)	3 (3.0; 4.0)
	6-10 years (n = 190)	3 (3.0; 3.0)
	11-15 years (n = 76)	4 (2.5; 4.5)
	16+ years (n = 88)	3 (2.0; 4.0)
**Living situation**	Living alone (n = 178)	3 (2.0; 3.0)
	Living with someone (n = 329)	3 (3.0; 4.0)
**Living area**	Rural (n = 247)	3 (3.0; 4.0)
	Urban (n = 195)	3 (3.0; 4.0)

strata with a small number of observations or deemed irrelevant to the analysis were excluded from the table or collapsed to other strata. Additionally, observations with missing information for the stratifying variable were not included in the respective strata. Categories were collapsed as follows: Education – “Secondary education” includes primary school, other education, and upper secondary; “Vocational education” includes vocational and short higher education ( < 3 years); “Medium education” includes intermediate higher education (3–4 years); “Higher education” includes higher education ( > 4 years). Work status – “Other” includes unemployed, on leave, or unspecified. Living situation – “Living alone” includes alone or with children; “Living with someone” includes partner, parents, roommates, or others. Living area – classified as urban or rural based on Danish postal codes.

### Participation in leisure-time activities within the last 4 weeks

Of the 563 participants, 524 completed the section of the questionnaire on leisure-time activities in the last 4 weeks. For the total participation level 19.1% 95%CI (15.9 to 22.7) participated <1 per week, 13.0% 95%CI (10.4 to 16.1) participated ~1 time per week, 22.1% 95%CI (18.8 to 25.9) participated ~2 times per week, 25.2 95%CI (21.7 to 29.1) participated ~3-4 times per week, 25.2% 95%CI (21.7 to 29.1) participated ~3-4 times per week, and 20.6% 95%CI (17.4 to 24.3) participated ≥5 times per week. For social activities, 37% had a participation frequency <1 per week, while 43% had participation <1 per week for non-social activities. The distribution of non-social and social participation levels can be found in Table 3. Table 4 shows leisure-time participation ( < 1 time per week over the past 4 weeks), stratified by demographic characteristics. A lower functional level to ambulate 100 meters was associated with a higher proportion of participation <1 per week, compared to those walking without aids, particularly regarding total participation. For social and non-social participation, this pattern was less consistent across strata, although an overall tendency was observed. A shorter education was also associated with a higher proportion of participation <1 per week, compared to those with longer education. No clear patterns were observed across work status; however, early retirees tended to report higher proportions of social participation <1 per week, compared to those who were employed. Similarly, living alone was associated with a higher proportion of social participation <1 per week, compared to living with someone. For biological sex, living situation, SCI characteristics, and time since injury and place of living differences in total, social, or non-social participation appeared minimal, and no consistent patterns were observed.

**Table 3:** Prevalence Proportion (PP, 95% CI) of participation levels non-social and social leisure-time participation within the last 4 weeks (n = 524).

Participation level	Non-social	Social
< 1 per week	43.5 (39.3; 47.8)	37.0 (33.0; 41.2)
~1 time per week	16.2 (13.3; 19.6)	22.7 (19.3; 26.5)
~2 times per week	23.1 (19.7; 26.9)	19.3 (16.1; 22.9)
~3-4 times per week	9.9 (7.6; 12.8)	13.7 (11.1; 17.0)
≥ 5 times per week	7.3 (5.3; 9.8)	7.3 (5.3; 9.8)

“< 1 per week”: less than once weekly. “~” indicates approximately.”≥ 5 times per week” means five or more times weekly.

**Table 4:** Prevalence Proportion Ratio (PPR), (95% CI) for < 1 per week of social, non-social, and total leisure-time participation within the last 4 weeks, stratified by demographic and SCI-related characteristics.

Characteristic	Groups	n	Total PPR (95% CI)	Non-social PPR (95% CI)	Social PPR (95% CI)
**Biological sex**	Men	344	1.22 (0.80, 1.87)	1.16 (0.87, 1.53)	1.23 (0.90, 1.67)
	**Women**	**180**	**1.00 (0.60, 1.66)**	**1.00 (0.72, 1.39)**	**1.00 (0.69, 1.44)**
**Age groups**	76+	82	2.10 (0.50, 8.92)	1.76 (0.70, 4.41)	1.35 (0.53, 3.44)
	61-75	211	1.39 (0.33, 5.74)	1.32 (0.54, 3.25)	1.00 (0.40, 2.47)
	46-60	156	1.25 (0.30, 5.27)	1.17 (0.47, 2.92)	1.19 (0.48, 2.97)
	31-45	60	1.25 (0.27, 5.71)	1.05 (0.40, 2.78)	1.00 (0.38, 2.66)
	**18-30**	**15**	**1.00 (0.14, 7.10)**	**1.00 (0.29, 3.45)**	**1.00 (0.29, 3.45)**
**Work Status**	Early Retirement	158	1.38 (0.82, 2.31)	1.24 (0.88, 1.74)	1.49 (1.03, 2.15)
	Retired	170	1.32 (0.79, 2.20)	1.31 (0.94, 1.83)	1.26 (0.86, 1.84)
	**Employed**	**160**	**1.00 (0.57, 1.74)**	**1.00 (0.70, 1.43)**	**1.00 (0.67, 1.50)**
	Other	18	0.71 (0.17, 3.00)	0.90 (0.39, 2.09)	1.70 (0.83, 3.47)
**Educational Level**	Secondary Education	90	2.84 (1.40, 5.79)	1.75 (1.12, 2.73)	1.56 (0.96, 2.56)
	Vocational Education	206	1.67 (0.84, 3.32)	1.26 (0.83, 1.91)	1.23 (0.78, 1.92)
	Medium Education	135	0.71 (0.31, 1.65)	0.84 (0.52, 1.35)	1.00 (0.61, 1.63)
	**Higher Education**	**80**	**1.00 (0.42, 2.40)**	**1.00 (0.60, 1.67)**	**1.00 (0.57, 1.74)**
**Living Situation**	Living alone	178	1.37 (0.92, 2.05)	1.02 (0.77, 1.34)	1.36 (1.02, 1.81)
	**Living with someone**	**333**	**1.00 (0.69, 1.45)**	**1.00 (0.79, 1.26)**	**1.00 (0.77, 1.30)**
**SCI characteristic**	**Paraplegia**	**256**	**1.00 (0.65, 1.55)**	**1.00 (0.76, 1.31)**	**1.00 (0.75, 1.34)**
	Tetraplegia	235	1.47 (0.98, 2.21)	1.13 (0.86, 1.48)	1.14 (0.85, 1.52)
**Functional Level**	Wheelchair & Assistance	87	4.78 (2.46, 9.28)	2.01 (1.37, 2.96)	1.84 (1.20, 2.81)
	Manual wheelchair	120	2.71 (1.36, 5.39)	1.27 (0.86, 1.90)	1.49 (0.98, 2.25)
	Walks with difficulty	140	2.60 (1.32, 5.11)	1.50 (1.04, 2.17)	1.46 (0.98, 2.18)
	**Walks without aids**	**156**	**1.00 (0.45, 2.23)**	**1.00 (0.67, 1.49)**	**1.00 (0.65, 1.53)**
**Years Since Injury**	0-2 years	7	N/A	0.32 (0.04, 2.32)	0.77 (0.19, 3.15)
	3-5 years	131	0.83 (0.49, 1.39)	0.88 (0.62, 1.24)	1.03 (0.72, 1.48)
	**6-10 years**	**192**	**1.00 (0.64, 1.56)**	**1.00 (0.74, 1.35)**	**1.00 (0.72, 1.39)**
	11-15 years	76	1.10 (0.62, 1.95)	1.13 (0.77, 1.66)	0.96 (0.62, 1.50)
	16+ years	88	1.01 (0.58, 1.76)	1.08 (0.74, 1.56)	1.01 (0.67, 1.53)
**Living area**	Rural	250	0.86 (0.57, 1.29)	1.03 (0.77, 1.38)	0.88 (0.65, 1.18)
	**Urban**	**196**	**1.00 (0.66, 1.52)**	**1.00 (0.74, 1.36)**	**1.00 (0.74, 1.36)**

Reference groups are shown in bold font. Strata with a small number of observations or deemed irrelevant to the analysis were excluded from the table or collapsed to other strata. Additionally, observations with missing information for the stratifying variable were not included in the respective strata. Categories were collapsed as follows: Education – “Secondary education” includes primary school, other education, and upper secondary; “Vocational education” includes vocational and short higher education ( < 3 years); “Medium education” includes intermediate higher education (3–4 years); “Higher education” includes higher education ( > 4 years). Work status – “Other” includes unemployed, on leave, or unspecified. Living situation – “Living alone” includes alone or with children; “Living with someone” includes partner, parents, roommates, or others. Living area – classified as urban or rural based on Danish postal codes.

### Organization of leisure-time activities

Of the leisure-time activities reported, 39% were self-organized, 29% were facilitated by commercial providers, 22% were organized by voluntary associations, and 13% were involved in other community-based activities.

### Motivation and barriers to participation in leisure-time activities

**Fig. 1:**
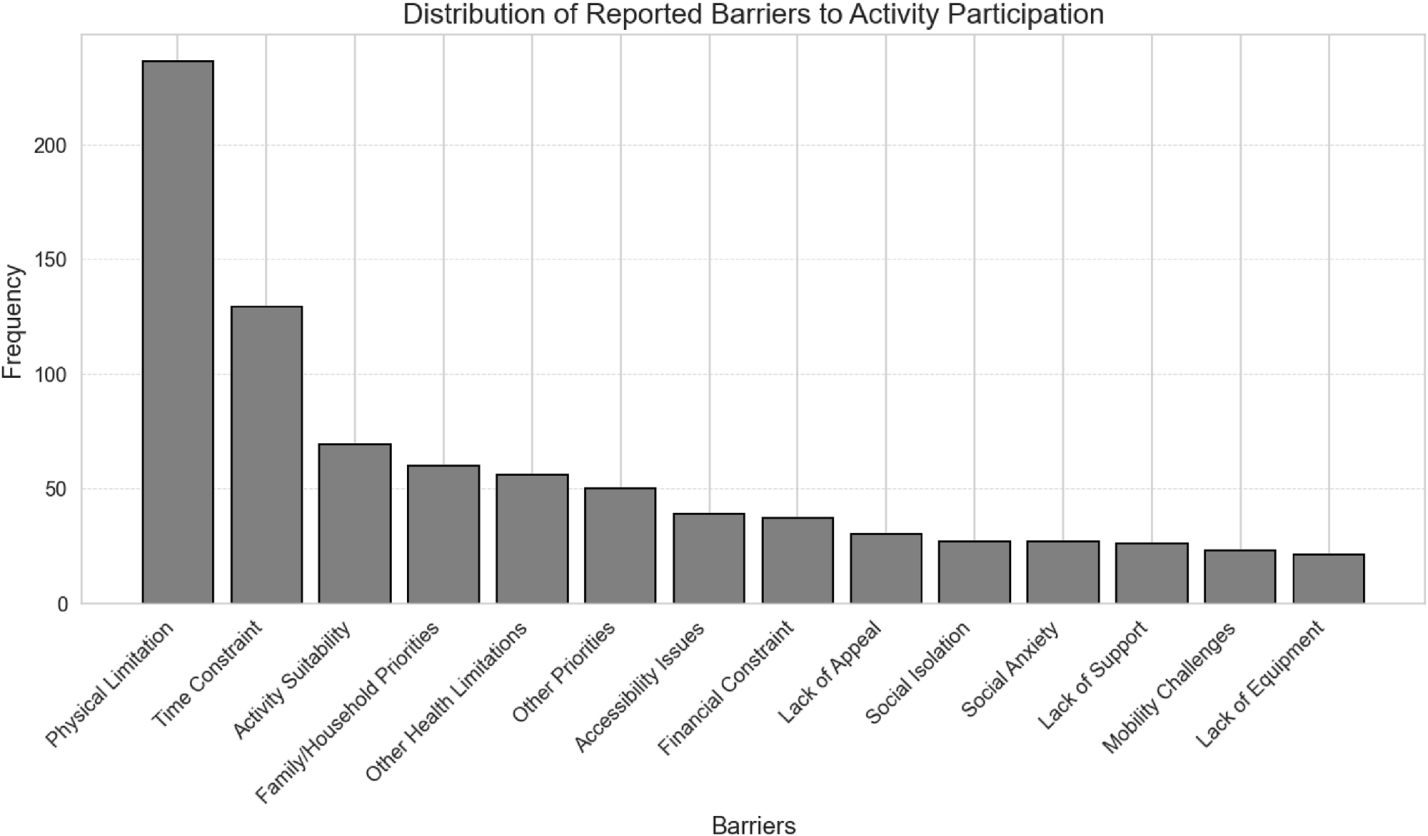
Frequency of barriers to participation in leisure-time activities (*n* = 439). *Note:* Barriers to participation included physical limitations (e.g., pain, fatigue, reduced function), time constraints (e.g., work obligations, limited availability), lack of suitable activities (e.g., not adapted to wheelchair use), and family responsibilities (e.g., childcare, household tasks). Other barriers were health conditions (e.g., heart disease, anxiety), accessibility or transport issues (e.g., inaccessible facilities), financial constraints (e.g., activity costs), limited interest, social isolation, insufficient support, mobility challenges, and lack of appropriate equipment (e.g., sports devices).

Of the 512 responses, 15% were not motivated to engage in additional leisure-time activities, 34% were somewhat motivated, 36% were moderately motivated, and 15% were highly motivated. The most frequent barriers to leisure-time participation were physical limitations, time constraints, and activity suitability. Figure 1 shows the distribution of these barriers.

## Discussion

This study describes participation in leisure-time activities among people living with SCI. The number of different leisure-time activities that people participated in within the last 12 months was approximately three, and this varied with age, work status, and functional level. Notably, the tendency of decreasing number of different activities varied with increasing age and lower mobility at 100 meters. Further, employed people participated in more different activities compared to retirees. Within the last 4 weeks, 19% of participants reported participation in leisure-time activities less than once a week. When separating social and non-social leisure-time activities, 44% reported participation less than once a week in non-social leisure-time activities and 37% reported participation in social leisure-time activities less than once a week. Participation less than once a week was more common in people with shorter education and people with lower physical functional levels. The organization of leisure-time activities was predominantly self-organized or facilitated by commercial providers, while voluntary associations and other community-based initiatives were less prevalent. The common barriers to leisure-time participation were physical limitations, time constraints, activity suitability, and competing priorities (e.g. work, family, household).

This is the first study to describe patterns of participation in leisure-time activities among people with SCI in Denmark. Previous studies of physical activity participation have been conducted on the Danish general population, which also included people living with various disabilities (e.g. cognitive, visual and auditory) [22, 26]. In the general Danish population, 10% of people were not physically active in leisure-time activities during the week [22], which was 3-5% in people with various disabilities [26]. The most comparable estimate is our total participation estimate for <1 per week time at 19% compared to 10% for the general population and 3-5% for the general population with disabilities. Our estimate suggests that people with SCI more frequently participate less than once a week, when compared to the general population. Further, people with SCI more frequently participate less than once a week, compared to people with various disabilities. However, several factors need to be considered when comparing results. For example, the estimates from the general population are based on a large cohort, and the classification of disability was vague, ranging from psychological to physical disabilities. The varying degrees of disability within the general population may not accurately represent specific subtypes of disability, such as SCI. Consequently, it is possible that these estimates reflect a population with different (likely higher) functional levels than those with SCI. Furthermore, differences in the definition of participation categories and differing inclusion criteria for activities, also affects comparability. Our estimate includes participation in non-physical leisure-time activities (e.g. creative activities, social activities), which increases our participation rate and excludes rehabilitation activities (e.g. physical therapy or craft therapy) which decreases the participation rate. For people with SCI, this reduced participation can lead to increased social isolation and fewer opportunities for meaningful social relations (connections) as well as affect essential emotional support, a sense of belonging, and mental stimulation, which are crucial for well-being [27, 28] and health-related quality of life.

Most existing literature focuses on physical activity and participation in leisure-time physical activities (LTPA) [29], making comparisons with our study challenging. Our research includes a wider definition of leisure-time activities, encompassing both physical and non-physical activities. However, a recent systematic review suggests that 27%-64% of middle/older aged adults participate in no LTPA [29]. This may align with our results when considering differences in age (older people in that study) and the inclusion of physically active and non-active activities in our study. The estimates of LTPA suggest that physical activity may not be suitable or appealing for everyone with SCI due to personal preferences or physical limitations. In such cases, all types of leisure-time activities, whether they involve physical activity or not, can still facilitate community and social engagement and contribute to overall well-being in everyday life. This highlights the importance of promoting diverse leisure-time activities to support the varied needs and preferences of people with SCI.

Gross-Hemmi et al. conducted a cross-sectional population-based study in Swiss people with SCI [30]. Participation in ADLs was measured using the Utrecht Scale for Evaluation of Rehabilitation-Participation (USER-P). The results showed that lower scores across all participation scales were associated with more severe SCI, comparable with our findings on the association between mobility and leisure-time participation. Additionally, they also found that lower levels of education were associated with lower participation, which was observed in the current study. Furthermore, higher age, being female, and not having a partner were associated with lower levels of participation which was not consistently observed in our study. Halvorsen et al. which also used the USER-P in Norwegian people with SCI, found that sociodemographic characteristics (e.g. employment and family income) were important for participation at all scales of USER-P [31], while disease severity (e.g. American Spinal Injury Association Impairment Scale, and time since discharge) was less important for participation. Though their assessment methods differ, their results align with ours, in that people with higher mobility and higher educational levels participate more frequently in leisure-time activities.

Participants reported physical limitations and activity suitability (nearby activities not in their preference) as frequent barriers for participation in leisure-time activities. These findings were similar to other studies of barriers to participation in leisure-time activities in persons with SCI [22, 32, 33]. Rehabilitation aims to facilitate the highest possible level of functioning within all domains of everyday living and further influence the environment and surroundings, so for example leisure-time activities outside the home become accessible. However, this process assumes that even though patients undergo rehabilitation, and often benefit from it, they must prepare for and cope with their newly acquired disability. They may not be able to engage in their previous leisure-time activities when they return home and may require alternative leisure-time activities. Most participants in this study reported that they were motivated to engage in more leisure-time activities. This could indicate a need to focus on helping people with disabilities engage in leisure-time activities in their local communities.

The organization of leisure-time activities showed similar patterns to the general population, where self-organized and commercial arrangements were more common than voluntary associations and other community-based initiatives [22]. Only 22% of the leisure-time activities reported in this study were organized by or performed with voluntary associations, which might indicate opportunities for helping more people engage in structured, low-cost, social and local leisure-time activities. To provide examples of initiatives, a systematic review identified various interventions aimed at increasing social participation for adults with traumatic brain injury [33]. These were Leisure Education Programs, Single Activity Group Leisure Interventions, and Multi-option Leisure Interventions. Specifically, Multi-option Leisure Interventions, which offer a variety of tailored activities to meet individual preferences and goals, may be relevant to people with SCI. Studies within the SCI population have focused on increasing physical activity [34], and further community integration. A scoping review on community integration recommended that goal setting and promoting self-efficacy should be included in programs, and people with lived experience of SCI should be involved in the development of these programs [35].

### Strengths and limitations

The cross-sectional survey-based design has several limitations. Firstly, the design only describes a specific time point and does not capture changes over time; moreover, the cross-sectional nature of the study does not allow for causal interpretation. Some leisure-time activities are prone to seasonal variations. Although the questionnaire did cover participation in activities within the last 12 months, there could be some recall bias in some respondents. The questionnaire was performed during Autumn, which might capture both outdoor and indoor activities.

We sampled 563/1259 of the identified eligible participants. In Denmark, there are approximately 9000 people living with SCI [36]. We included approximately 6% of the target population, we were uncertain if the population is representative. We used digital mail as the only contact source which is only available to people with Danish citizenship. Further, approximately 10% of the study population did not have digital mail and could not receive the invitation to participate. Non-responders received two reminders through digital mail, but they were not contacted using other approaches (e.g. phone call, text messages). This may have resulted in the inclusion of higher functioning and younger people. All participants were offered assistance to complete the questionnaire (by phone call), but only one participant used this opportunity. People who participate in leisure-time activities may also be more likely to complete the survey. The above-mentioned potential selection biases may have overestimated participation rates. Future studies should focus on improving response rates by using several contact methods and invitation sources to secure a more representative study population and a higher response rate. Though the overall participation rate may be affected by selection bias, we obtained participants in the groups used for stratification.

We recognize that participation research has increasingly shifted toward theory-based approaches using standardized measures to examine predictors, outcomes, and mechanisms in order to inform targeted interventions. Our study differs in that it is primarily descriptive and based on the frequency of engagement in a wide range of leisure-time activities. While this approach does not capture the quality or subjective experience of participation, it provides a straightforward and comparable measure that can highlight overall patterns in a large population.

The aggregation of participation across activities and the categorization of participation frequency offer a practical estimate of engagement. The graded scoring system reduces the risk of overrepresenting individuals with very high participation, thereby allowing clearer differentiation between participants. While this method supports a transparent classification of engagement levels, it does not fully reflect the intensity or variability of participation for all individuals. Consequently, the resulting scores are best suited for relative comparisons across participants rather than precise measurement.

In addition, stratifying participation proportions across key variables provides an accessible and interpretable method for examining subgroup differences in descriptive analyses. This approach avoids heavy reliance on parametric assumptions and can reveal heterogeneity between strata. However, it is less suitable in contexts involving multiple confounding variables or small subgroup sizes, as this can produce unstable or unreliable estimates. Unlike multivariable regression techniques, stratified analyses cannot account for the simultaneous effects of several covariates, which may obscure independent associations. Nevertheless, by applying these methods to frequency-based data, our study contributes useful descriptive evidence and may help identify subgroups at greater risk of limited participation, thereby guiding the development of future theory-based interventions.

Participation in leisure-time activities and the organization of these may vary across national regions and countries. In Denmark leisure-time activities have historically been organized by national and local volunteer associations, such as sports and hobby communities, which might be different in other countries where other types of organization might be more common (e.g. commercial or school-based). The generalizability of the results from this study may be affected by other demographic patterns where access to e.g. education may be different from the Danish education system which is free of charge, and the economy and culture for engaging in leisure-time activities may play a more vital role.

### Clinical implications

The proportions of participation in leisure-time activities less than once a week, among people with SCI might describe a need for targeted interventions to promote engagement. Rehabilitation strategies should address barriers such as physical limitations and activity suitability by providing accessible options, strengthen patient confidence and support self-efficacy. Diverse leisure-time activities should be promoted, and systematic integration into care plans is essential for ongoing engagement. While the field of participation research has increasingly advanced toward theory-based approaches using standardized questionnaires to examine predictors, outcomes, and intervention strategies, we believe that descriptive studies such as ours may still provide valuable insights. In particular, identifying patterns linked to sociodemographic factors can help highlight subgroups who might benefit from tailored interventions to further support meaningful participation in everyday life.

## Conclusion

This study suggests that approximately 20% of people living with SCI in Denmark participate in leisure-time activities less than once a week. Further, 37% participated in social leisure-time activities less than once a week. People participating less than once a week had shorter education and more difficulties with ambulation at 100 meters. Barriers such as physical limitations, time constraints, and activity suitability were most prevalent. More than half the study population were motivated to engage in more leisure-time activities than they currently do. The organization of activities is predominantly self-organized or commercially facilitated. Overall, the results may indicate potential areas for intervention to enhance general participation in leisure-time activities, preferably through volunteer associations.

### Declaration of generative AI in scientific writing

During the preparation of this work the author(s) used ChatGPT to rephrase sentences and increase understandability. Further, ChatGPT was used to generate and debug code for data management and analysis in Python. After using this tool/service, the author(s) reviewed and edited the content as needed and take(s) full responsibility for the content of the publication.

## Supplementary information

S.1. Supplementary material

S.2. Supplementary material

## Data Availability

The datasets generated during and/or analyzed during the current study are not publicly available due to the nature of the personal health data. Any questions regarding the dataset will be answered to the best of our ability by the corresponding author upon reasonable request.
