# Supplementary material for: Participation in leisure-time activities among people living with Spinal Cord Injuries. A cross-sectional survey
Source: Spinal Cord Ser Cases. 2025 Oct 23;11:27. doi: 10.1038/s41394-025-00722-0 (PMC12550068; doi:10.1038/s41394-025-00722-0)
Supplement: Supplementary file 1 — S.1. Supplementary material [file 41394_2025_722_MOESM1_ESM.docx]

**Figure S.1. - Proportion of people who participated in individual activities within the last 12 months. (n=535)**


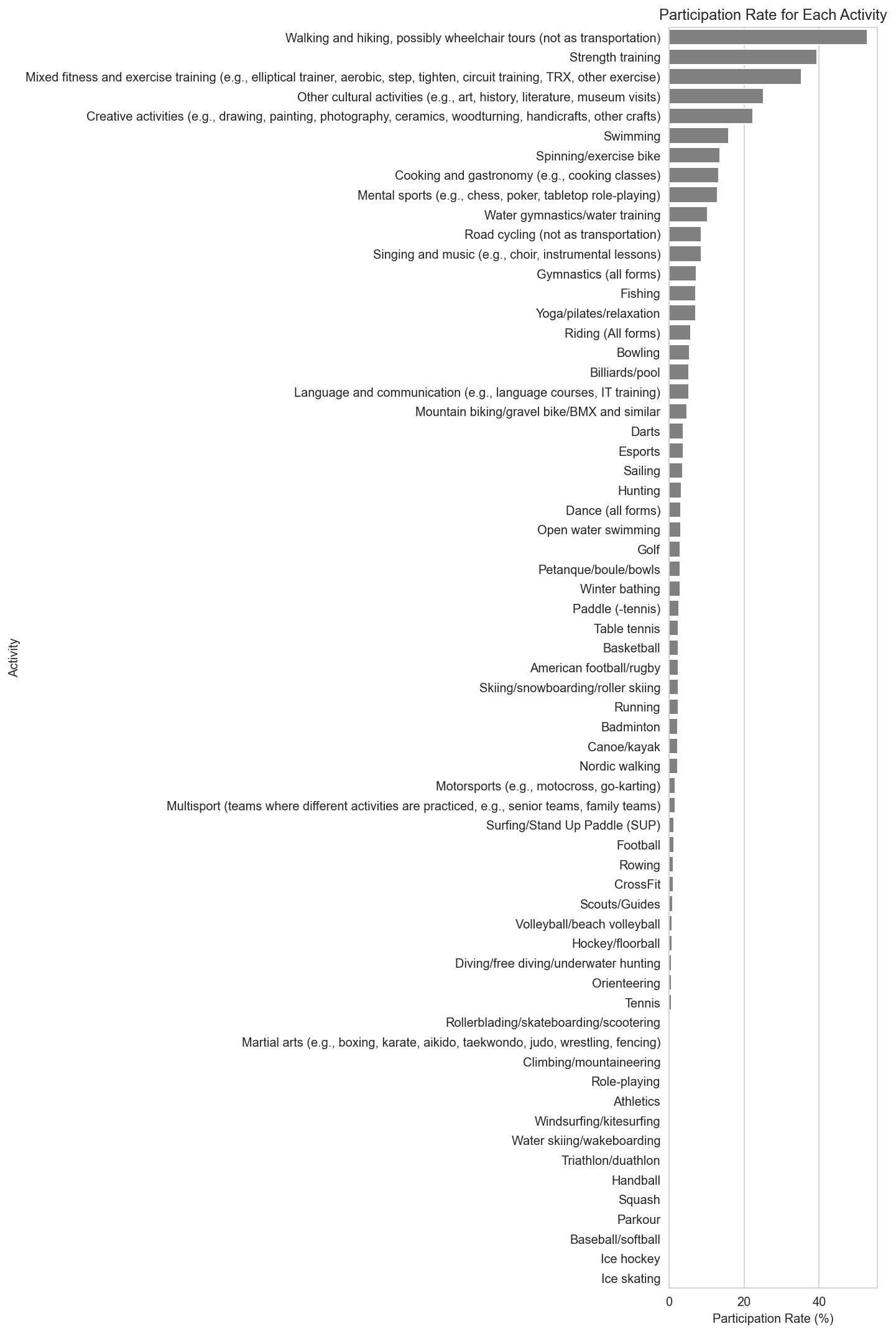


| **Note:** Figure A.1 presents the percentage of respondents who reported participating in each activity within the past 12 months. Activities are displayed in descending order of participation rate. It should be noted that the categorisation does not differentiate between activities performed in conventional forms and those adapted for wheelchair users or individuals with functional impairments. Walking and hiking (which may include the use of mobility aids), strength training, and mixed fitness activities (e.g., elliptical training, step aerobics) were among the most commonly reported activities. Less frequently reported were high-impact sports and structured team activities. |
| --- |
