# Supplementary material for: Participation in leisure-time activities among people living with Spinal Cord Injuries. A cross-sectional survey
Source: Spinal Cord Ser Cases. 2025 Oct 23;11:27. doi: 10.1038/s41394-025-00722-0 (PMC12550068; doi:10.1038/s41394-025-00722-0)
Supplement: Supplementary file 2 — S.2. Supplementary material [file 41394_2025_722_MOESM2_ESM.docx]

**S.2. Activity participation scoring and categorization system**

**Overview**

The system quantifies participation in various activities based on reported frequency, generating a total participation score that is then categorized into engagement levels. The approach ensures a gradual and proportional increase in scores, avoiding overrepresentation of frequent participation while maintaining distinctions between different engagement levels.

**Scoring**

Each reported activity frequency is assigned a numerical score based on its contribution to overall engagement:

| **Reported Frequency** | **Assigned Score** | **Rationale** |
| --- | --- | --- |
| No participation ("non", "less often") | 0 | No engagement |
| 1-3 times per month | 0.25 | Minimal engagement, but participation |
| 1 time per week | 0.75 | Low but regular participation |
| 2 times per week | 1.5 | Moderate participation |
| 3-4 times per week | 2.5 | High participation |
| 5 or more times per week | 3.5 | Very high and frequent participation |

The scoring ensures progression rather than abrupt jumps, making the total score more representative of actual behavior.

**Summing**

For each individual, the total participation score is calculated by summing the scores from all activities they engage in.

**Categorizing of total participation, non-social and social**

To classify individuals into distinct engagement groups, we applied the following cut-offs to the summed participation scores:

| **Summed Score Range** | **Participation Category** |
| --- | --- |
| 0 – 0,74 | <1 time per week |
| 0.75 – 1.5 | ~1 time per week |
| 1.6 – 3.5 | ~2 times per week |
| 3.6 – 6.5 | ~3-4 times per week |
| ≥6.5 | ≥5 times per week |

This classification ensures that the final categories reflect different levels of engagement across multiple activities.

**Example of cases**

To illustrate how the system works, here are example cases of individuals with different participation levels:

| **Case** | **Activities included** | **Summed Score** | **Category** |
| --- | --- | --- | --- |
| Case 1 | Less often, 1-3 times/month, 1 time/week | 1.0 | ~1 time per week |
| Case 2 | 2 times/week, 1 time/week | 2.25 | ~2 times per week |
| Case 3 | 3-4 times/week, 2 times/week, 1-3 times/month | 4.25 | ~3-4 times per week |
| Case 4 | 5+ times/week, 3-4 times/week, 2 times/week | 7.5 | ≥5 times per week |
| Case 5 | 5+ times/week, 5+ times/week | 7.0 | ≥5 times per week |

**Benefits:**

- The system prevents inflated scores while maintaining meaningful differences in participation levels.
- The score increments ensure a smooth transition between categories.
- The final classification aligns well with real-world engagement patterns, making it easy to interpret.

The system is intended to provide a clear and structured approach for measuring activity participation across multiple domains.
